# Supplementary material for: Alpha/Beta Hydrolase Domain-Containing Protein 2 Regulates the Rhythm of Follicular Maturation and Estrous Stages of the Female Reproductive Cycle
Source: Front Cell Dev Biol. 2021 Sep 8;9:710864. doi: 10.3389/fcell.2021.710864 (PMC8455887; doi:10.3389/fcell.2021.710864)

Supplemental Fig. S1

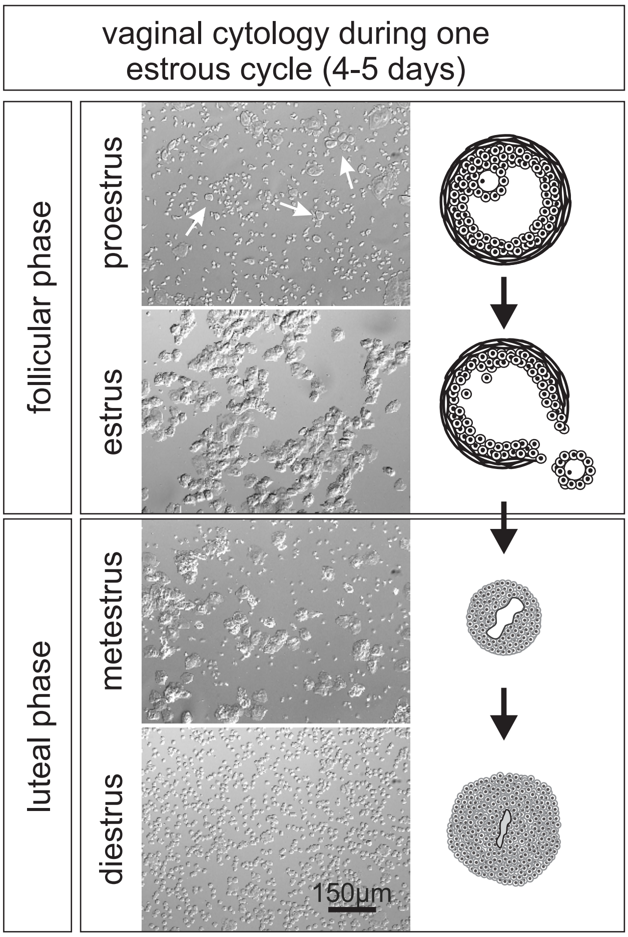

Suppplemental Fig. S2

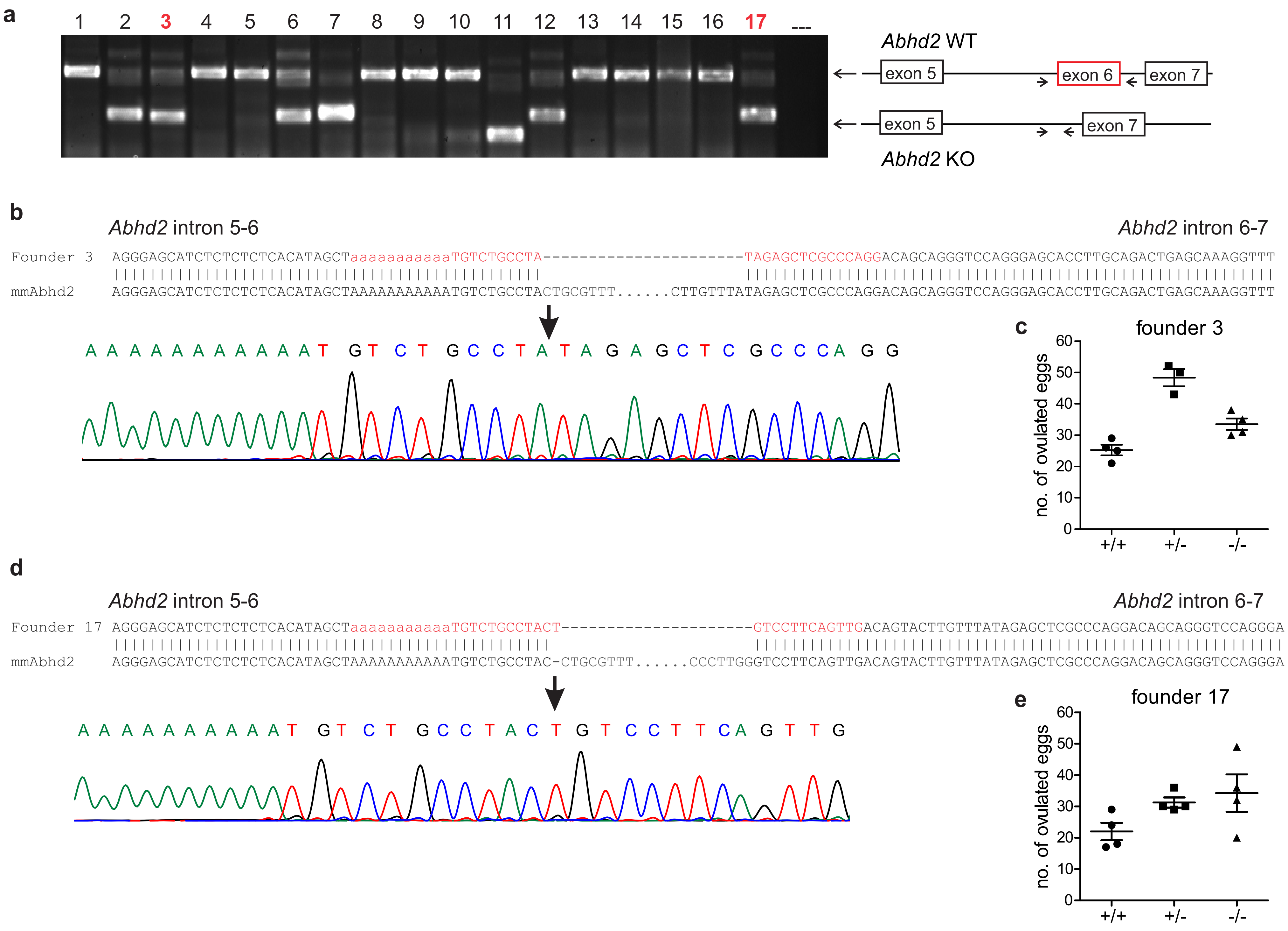

Suppplemental Fig. S3

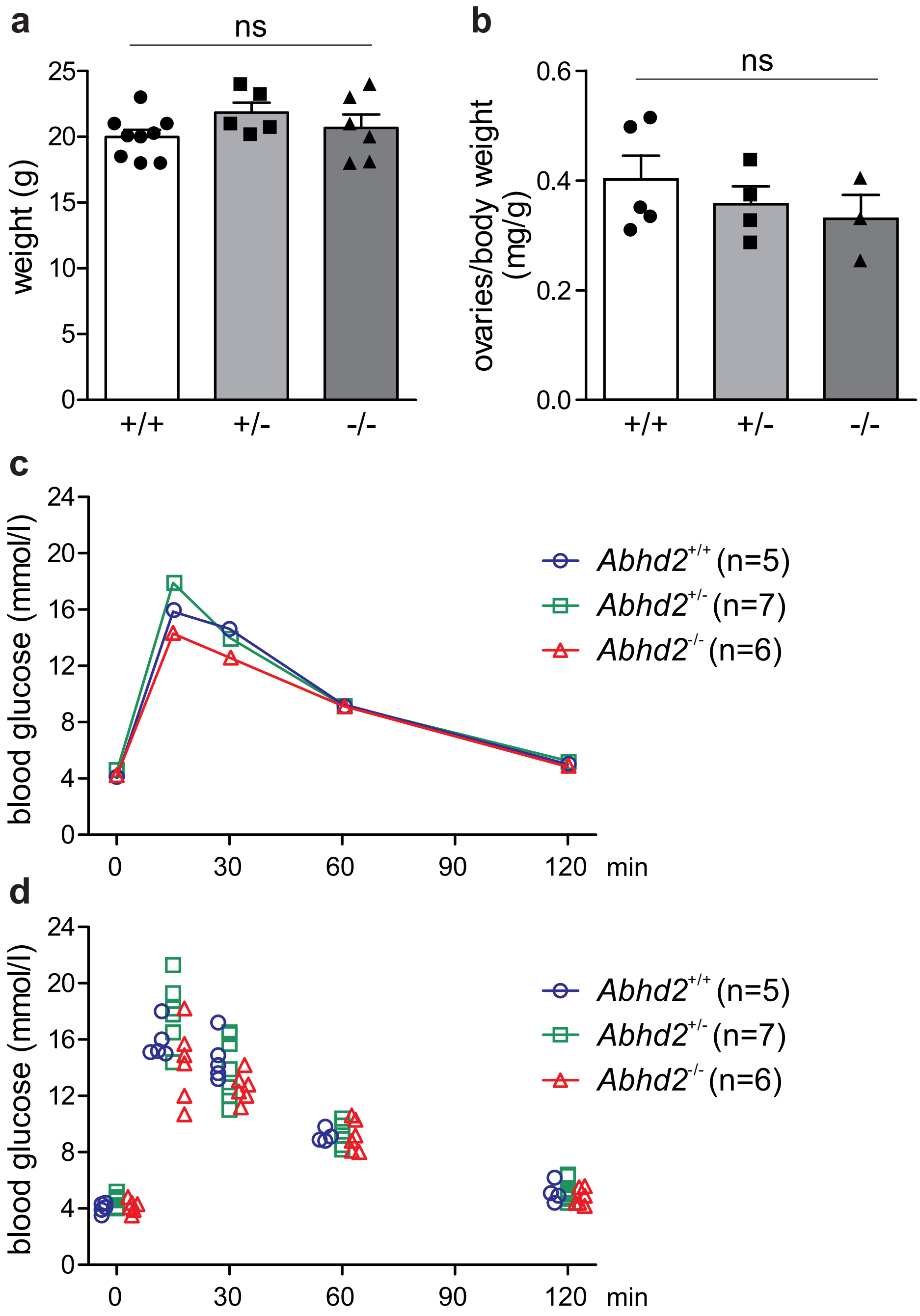

Suppplemental Fig. S4

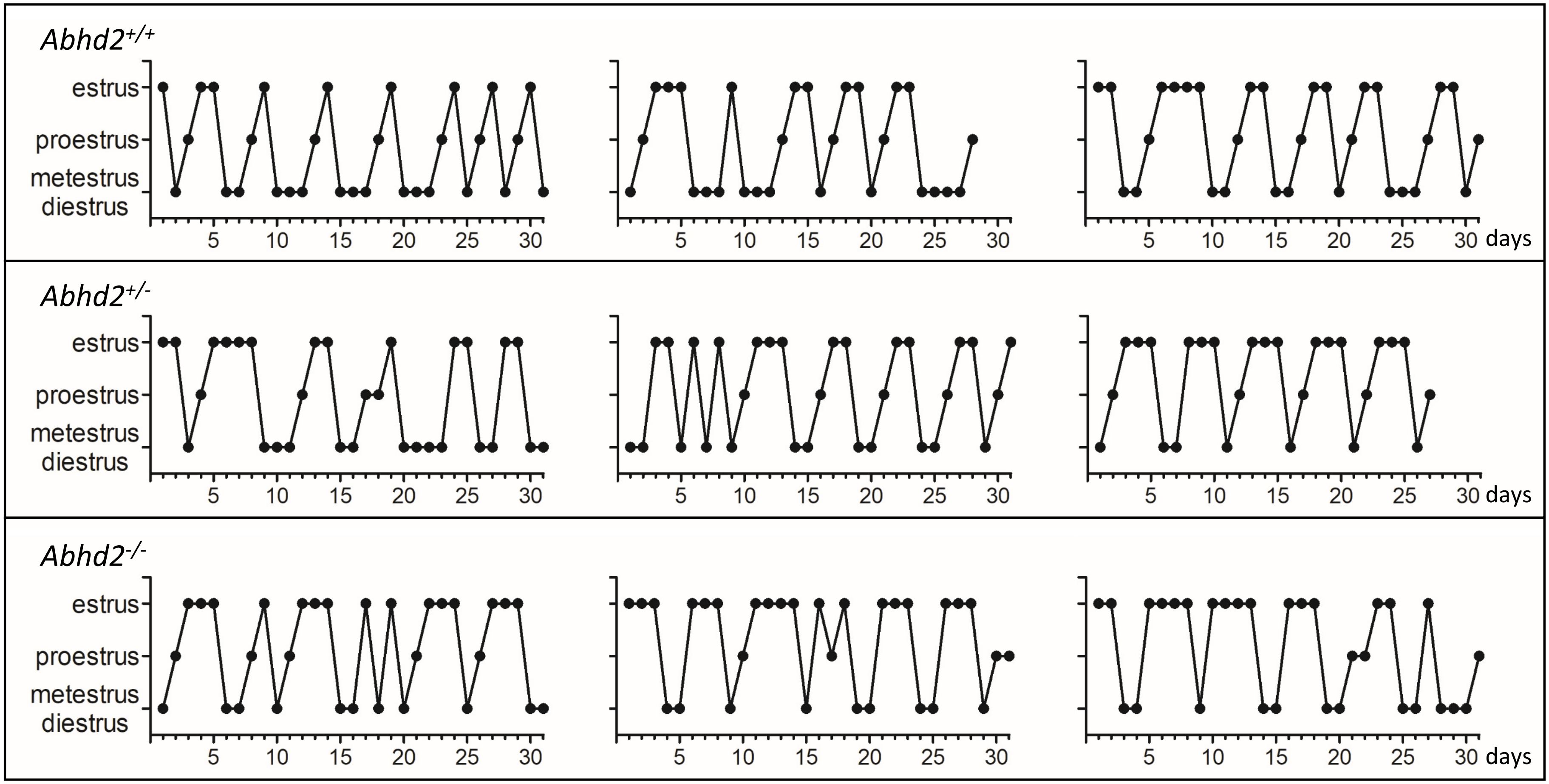

Suppplemental Fig. S5

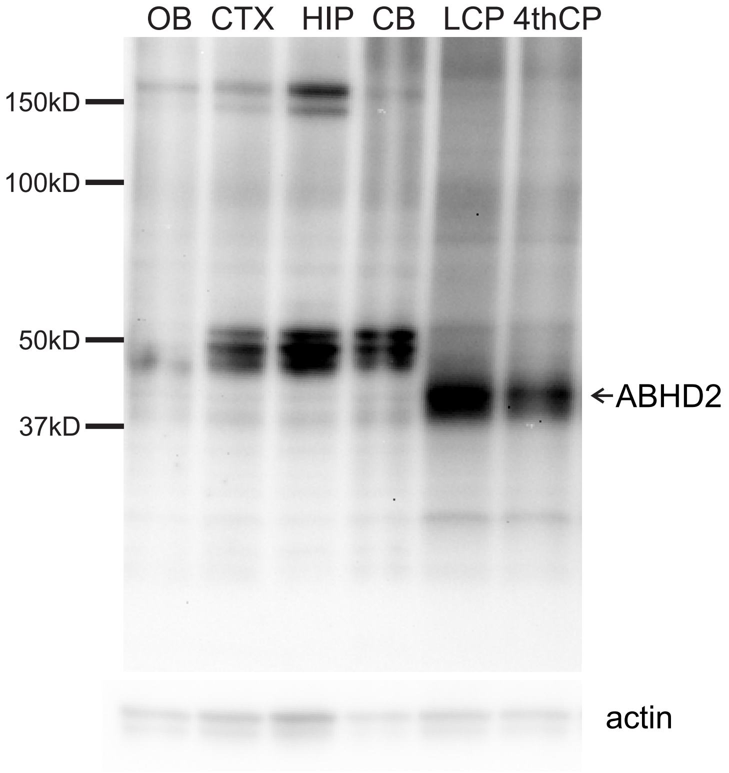

Supplement: Supplementary Figure 1 — Estrous cycle of mice. Smears from vaginal lavage of mice show the distinct cell types of different estrous stages. Proestrus shows multiple nucleated cells (arrows) while the estrus sample only displays cornified epithelial cells. When the mouse enters the luteal phase, leukocytes start to appear and at diestrus they are the dominant cell type in the smear. At proestrus the antral follicle is formed, as depicted in the drawing. The mature oocyte is ovulated at estrus after which the remaining follicle goes through luteinization and forms corpus luteum (CL). Active secretion of progesterone from CL peaks at diestrus followed by luteolysis of the CL cells and resumption of proestrus. [file Data_Sheet_1.PDF]
