# Supplementary material for: Alpha/Beta Hydrolase Domain-Containing Protein 2 Regulates the Rhythm of Follicular Maturation and Estrous Stages of the Female Reproductive Cycle
Source: Front Cell Dev Biol. 2021 Sep 8;9:710864. doi: 10.3389/fcell.2021.710864 (PMC8455887; doi:10.3389/fcell.2021.710864)
Supplement: Supplementary file 2 [file Table_1.docx]

**Supplementary Table 1**

| **Gene** |  | **5’- primer – 3’ sequence** | **Tm ˚C** |
| --- | --- | --- | --- |
| abhydrolase domain containing 2 | Abhd2 e3 Fw | CCGTAGCTGCCGTTCTCTAC | 61 |
|  | Abhd2 e4 Re | CTCCCCATCTTCCCATACAA |  |
|  | Abhd2 e5 Fw | GAATTGCCAACCACAGCGAG | 61 |
|  | Abhd2 e6 Rev | AGCTGGGTCTGGGGATATGT |  |
| follicle stimulating hormone receptor | Fshr Fw | TGTTTTCCAGGGAGCCTCTG | 61 |
|  | Fshr Rev | AGCAGTGACTGGGGTAGGTA |  |
| cytochrome P450 family 11 subfamily A member 1 | Cyp11a1 Fw | ATTACCGAGATGCTGGCAGG | 60 |
|  | Cyp11a1 Rev | GTGTCTCCTTGATGCTGGCT |  |
| cytochrome P450 family 17 subfamily A member 1 | Cyp17a1 Fw | CAATGACCGGACTCACCTCC | 60 |
|  | Cyp17a1 Rev | CCTTCGGGATGGCAAACTCT |  |
| vascular endothelial growth factor A | Vegfa Fw | CGATTGAGACCCTGGTGGAC | 61 |
|  | Vegfa Rev | GCTGGCTTTGGTGAGGTTTG |  |
| nerve growth factor | Ngf Fw | TGTGCCTCAAGCCAGTGAAA | 60 |
|  | Ngf Rev | CACTGAGGTGAGCTTGGGTC |  |
| neurotrophic receptor tyrosine kinase 1 | Ntrk1 Fw | CATCGTGCGCTTCTTTGGAG | 60 |
|  | Ntrk1 Rev | CAGCAGCTTTGCATCAGGTC |  |
| nerve growth factor receptor (TNFR superfamily, member 16) | Ngfr Fw | CCGCTGACAACCTCATTCCT | 60 |
|  | Ngfr Rev | TGTCGCTGTGCAGTTTCTCT |  |
